# Three important short-chain fatty acids (SCFAs) attenuate the inflammatory response induced by 5-FU and maintain the integrity of intestinal mucosal tight junction

**DOI:** 10.1186/s12865-022-00495-3

**Published:** 2022-04-21

**Authors:** Xi Yue, Sun Wen, Ding Long-kun, Yan Man, Sun Chang, Zhang Min, Li Shuang-yu, Qian Xin, Ma Jie, Wu Liang

**Affiliations:** 1 Medical College of Jiangsu University, Zhenjiang, 212013 Jiangsu People’s Republic of China; 2 Department of Critical Care Medicine, Jurong Hospital Affiliated to Jiangsu University, Zhenjiang, 212400 People’s Republic of China

**Keywords:** Short-chain fatty acids, 5-Fluorouracil, Inflammation, Intestinal mucosal barrier

## Abstract

**Background:**

5-Fluorouracil (5-FU) is a used chemotherapy drug for cancer, and its main side effect is intestinal mucositis which causes chemotherapy to fail. It was known that short-chain fatty acids (SCFAs) can inhibit immune cell release of various proinflammatory factors and inhibit excessive intestinal inflammation. However, the inhibitory effect of SCFAs on 5-FU-induced intestinal mucositis is still unclear.

**Results:**

To simulate the effects of SCFAs on immune and intestinal epithelial cells, the cells (THP-1 cells and Caco-2 cells) were pretreated with sodium acetate (NaAc), sodium propionate (NaPc) and sodium butyrate (NaB), then inflammation was induced by 5-FU. The expressions of reactive oxygen species (ROS), Beclin-1, LC3-II, NF-κB p65, NLRP3 inflammasome, proinflammatory/anti-inflammatory cytokines and mucosal tight junction proteins were determined. In our results, the three SCFAs could inhibit ROS expressions, NLRP3, Caspase-1, IL-1β, IL-6, IL-18, Beclin-1 and LC3-II, when induced by 5-FU. In a 5-FU-induced chemoentermuctis mouse model, *Lactobacillus rhamnoides* can increase the concentrations of three SCFAs in faeces and increase the concentrations of IL-1β, IL-6 and IgA in serum, and decrease the expressions of NLRP3 and IL-17 in spleen cells. The expressions of ZO-1 and Occludin in intestinal mucosa were significantly increased.

**Conclusions:**

These results indicated that the three SCFAs can effectively suppress the inflammation of THP-1 cells and Caco-2 cells and maintain tight junction integrity in intestinal mucosal epithelial cells.

## Highlights

- The three main SCFAs inhibit the expression of reactive oxygen expression, NLRP3 inflammasome, and proinflammatory cytokines.
- The three main SCFAs can reduce the autophagy of THP-1 cells to maintain host innate immunity.
- The three main SCFAs protect the integrity of the mucosal barrier of intestinal epithelial cells by increasing the expression of mucin MUC2 and tight junction protein.
- Increasing the content of three SCFAs in the mouse intestine by *Lactobacillus rhamnosus* can significantly protect the integrity of tight junctions between intestinal mucosal epithelial cells and improve the serum sIgA content to protect the intestinal mucosal immune barrier.

## Introduction

Short-chain fatty acids (SCFAs) are produced by indigestible dietary fiber fermented by some anaerobes in the intestine. These SCFAs are critical regulators of the "intestinal flora-immune axis". SCFAs are constituted with fewer than six carbon atoms. Among these SCFAs, acetate, propionate, and butyrate account for more than 90% of the total SCFAs which are usually present in the intestinal tract in an ionic state [1, 2]. SCFAs can promote the proliferation of intestinal mucosal epithelial cells, prevent intestinal mucosal epithelial cell atrophy to maintain the normal mucosal barrier, and inhibit the production of proinflammatory cytokines; thus reduce the overall inflammatory injuries. Clinical studies show that prognosis among patients with colitis can be improved by increasing dietary fibre or SCFAs intake. However, the exact mechanism is still unclear [3].

5-Fluorouracil (5-FU) is a commonly used tumor chemotherapy drug that inhibits tumor cell proliferation by interfering with tumor cell DNA synthesis and is widely used to treat various cancers [4, 5]. However, 40–80% of patients develop intestinal mucositis, which includes the symptoms such as indigestion, diarrhea, and dehydration, during the treatment, and shows resistance to chemotherapy [6]. Previous studies have shown that DNA damage, production of reactive oxygen species (ROS) and activation of NLRP3 inflammasome are key processes which induce the expression of proinflammatory cytokines (such as IL-1β, TNF-α, etc.) and the intestinal mucositis [7]. However, the mechanism of SCFAs inhibiting 5-FU-induced inflammation and maintaining the integrity of the mucosal barrier is not well-documented. Therefore, we designed this study to investigate the effects and underlying mechanism of three major SCFAs, namely acetate, propionate, and butyrate, on 5-FU-induced inflammatory response and intestinal mucosal barrier integrity.

## Materials and methods

### Cell culture and treatment

Human mononuclear macrophage (THP-1) cells and human colorectal adenocarcinoma (Caco-2) cells were purchased from Shanghai Cell Bank. THP-1 cells were cultured in RPMI 1640 medium containing 10% fetal bovine serum, and Caco-2 cells were cultured in DMEM medium containing 10% fetal bovine serum. Both of the cells were used in this study after stable passage to the sixth generation in the environment of 37 °C and 5% CO_2_. The cells in the logarithmic growth phase were inoculated in a 6-well plate with 1 × 10^6^ cells in each well. The normal control group (NC), 5-FU group (5-FU), 5-FU + sodium acetate group (NaAc), 5-FU + sodium propionate group (NaPc) and 5-FU + sodium butyrate group (NaB) were set up in the experiment. The cells in the NC group were not do any treatment. The NaAc group, NaPc group and NaB group cells were pretreated with 100 μmol/L NaAc, NaPc, NaB for 24 h, respectively. The cells in the 5-FU group, NaAc group, NaPc group and NaB group were treated with 5-FU for 24 h. The concentration of 5-FU was 2.5 mmol/L in THP-1 cells and 5 mmol/L in Caco-2 cells. All methods were carried out in accordance with relevant guidelines and regulations.

### Cell dsDNA detection

The supernatant of THP-1 cells was collected after treated by 5-FU for 24 h. The cell supernatant was centrifuged at 4 °C for 10 min. The concentration of dsDNA was determined by the dsDNA HS Assay kit (Qcbio Science &Technologies Co., Ltd, China), and the fluorescence intensities of 480 nm and 520 nm were detected the concentration of dsDNA was calculated following the instruction.

### Reactive oxygen species (ROS) detection

The concentration of ROS in cells was detected by a 2-dichlorofluorescein yellow diacetate (DCFH-DA) probe kit (Beyotime Biotechnology, China). The serum-free medium diluted DCFH-DA at the ratio of 1: 1000 to the final concentration of 10 μmol/L. The cells were resuspended in diluted DCFH-DA probe solution, incubated in a cell incubator at 37 °C for 20 min, and mixed upside down every 5 min so that the probe was in complete contact with the cells. The serum-free cell culture medium was washed 3 times to remove the DCFH-DA probe that did not enter the cell, and then resuspended the cells were resuspended with aseptic PBS buffer for detection following the kit instruction.

### Western blotting

The total cell protein was prepared by RIPA lysate (Beyotime Biotechnology, China). The cytoplasmic and nuclear proteins were separated by the nuclear and cytoplasmic protein extraction kits (Beyotime Biotechnology, China). The samples were electrophoretic by SDS-PAGE, transferred to PVDF membrane, sealed by 5% skimmed milk powder and incubated with the corresponding first antibody at 4 °C overnight, then incubated with a second antibody for 1 h. The ECL chemiluminescence kit was used (Millipore, USA), and the results were analyzed by Image J software. The β-actin was used as the reference for the expression of total cell protein and plasma protein analysis, and the Histone H1 was used as the reference for the expression of nuclear protein analysis. All experiments were repeated 3 times.

### Animal experiments

All male C57BL/6 mice (6–8 weeks, 20–25 g) were purchased from the animal experiment centre, Jiangsu university. All the animal experiment methods in our study were in accordance with ARRIVE guidelines and approved by the management committee and used of laboratory animals, Jiangsu University (No. UJS-IACUC-AP-2020032009). All the animals were randomly divided into three groups (n = 10), including the normal control group (NC), LGG group (LGG), 5-FU group (5-FU), LGG + 5-FU group (LGG + 5-FU) and intestinal flora disorder group (disorder + 5-FU). The mice in the LGG + 5-FU and LGG groups were fed 200 μL of *Lactobacillus rhamnosus* every day (2 × 10^9^ CFU/mL). On the 16th day, the disorder group mice were fed with 300 μL of the antibiotic mixture (containing 0.08 g/mL gentamicin and 0.05 g/mL cefradine) to induce intestinal flora disorder. The oral antibiotics were fed once a day for 3 days. On the 19th day, the mice in the 5-FU group, LGG + 5-FU group and disorder + 5-FU group were intraperitoneally injected 5-FU (30 mg/kg) for 5 days to induce intestinal mucositis. The NC group mice were normal breeding without any treatment. After 5-FU inducing for 24 h, all the mice were sacrificed by euthanasia method, and the serum, faeces, spleen and intestine were collected. Each group of mice was weighed for 7 consecutive days from 2 days before the induction of intestinal mucositis, and the weight changes were plotted.

### SCFAs determination

The concentrations of acetic acid, propionic acid and butyrate acid in faeces were determined by gas chromatography in the Testing Center of Yangzhou University. The faeces samples were mixed with distilled water in a ratio of 1:5, and the supernatants were collected by centrifugation at 4 °C at 12,000 rpm for 10 min. Finally, replenish the volume to 1 mL. The concentrations of acetic acid, propionic acid and butyrate acid were determined by gas chromatography [8].

### Immunohistochemical experiments

Wu [9] and Wang's [10] reporting methods were used for reference and slightly modified. Paraffin-embedded, 3 μm tissue sections were mounted onto SuperForst slides (SuperFrost Plus, Gerhard Menzel GmbH, Braunschweig, Germany), deparaffinized in xylene and ethanol of graded concentrations. For antigen retrieval, the slides were treated in a microwave oven in a solution of TRS for 30 min (2 × 6 min 360 W, 2 × 5 min 180 W, 2 × 4 min 90 W). After cooling down at room temperature, they were transferred to 0.3% hydrogen peroxide in methanol for 30 min to block endogenous peroxidase activities. Sections were rinsed with Tris-buffered saline and incubated with primary antibodies against ZO-1 (1:100, BOSTER, China) and Occludin (1:100, BOSTER, China). A biotinylated anti-rabbit secondary antibody was used at a dilution of 1:200. Detection was done with the ABC peroxidase system. DAB liquid was the peroxidase substrate.

### Enzyme-linked immunosorbent assay (ELISA)

The expression levels of IL-1β, IL-6 and IgA in the serum of mice were detected by ELISA kit (Meimiang, Jiangsu, China), and the absorbance of 450 nm was detected by enzyme labelling instrument. The standard curves of various cytokines were drawn, and the cytokines' contents in each group's serum were calculated.

### Real-time PCR (RT-PCR)

The method reported by Mehta was followed in our experiment [11]. Trizol reagent (Vazyme Biotechnology, China) was used to extract total RNA from cells and tissues. A reverse transcription kit (Vazyme Biotechnology, China) was used to reverse the total RNA cDNA. The primers used in this experiment are listed in Table 1. The Real-time PCR mixtures consisted of 5 μL cDNA corresponding to ~ 600 ng total RNA, 0.1 μM of primers and AceQ qPCR SYBR Green Master Mix (Vazyme, Najing, China) in a final volume of 25 μL. The thermal profile of the Real-time PCR procedure (Bio-rad CFX96 Real Time System, USA) repeated for 40 cycles including 95 °C for 1 min, 10 s denaturation at 95 °C and 40 s annealing at 55 °C. The 2^−△△Ct^ was used to calculate the relative gene expression. GAPDH and β-actin were used as internal references [11].

**Table 1:** The primers used in this experiment

Gene	Forward primer (5′→3′)	Reverse primer
Human NLRP3	AACAGCCACCTCACTTCCAG	CCAACCACAATCTCCGAATG
Mouse NLRP3	ATTACCCGCCCGAGAAAGG	CATGAGTGTGGCTAGATCCAAG
Human Caspase-1	GCACAAGACCTCTGACAGCA	TTGGGCAGTTCTTGGTATTC
Human IL-1β	CCTGTCCTGCGTGTTGAAAGA	GGGAACTGGGCAGACTCAAA
Human IL-6	CCTTCGGTCCAGTTGCCTTCT	GAGGTGAGTGGCTGTCTGTGT
Human IL-10	TCTCCGAGATGCCTTCAGCAGA	TCAGACAAGGCTTGGCAACCCA
Mouse IL-10	GAAGCTCCCTCAGCGAGGACA	TTGGGCCAGTGAGTGAAAGGG
Mouse IL-17	TTTAACTCCCTTGGCGCAAAA	CTTTCCCTCCGCATTGACAC
Human IL-18	TCTTCATTGACCAAGGAAATCGG	TCCGGGGTGCATTATCTCTAC
Human Occludin	CTTCCAATGGCAAAGTGAATGAATGAC	TACCACCGCTGCTGTAACGAG
Human ZO-1	GAGCCTAATCTGACCTATGAACC	TGAGGACTCGTATCTGTATGTGG
Human MUC2	CTTATCTGCTGTGTCCTGAA	AAGTCTCGTTCTCCTGTCT
Human GAPDH	CATCACTGCCACCCAGAAGACTG	ATGCCAGTGAGCTTCCCGTTCAG
Mouse β-actin	ATGACCCAAGCCGAGAAGG	CGGCCAAGTCTTAGAGTTGTTG

### Statistical analysis

Image J Software and GraphPad Prism8.0 analysis software were used for image analysis and data processing after protein development. SPSS 22.0 software was used for statistical analysis, and all data were expressed. Single-factor analysis of variance (ANOVA) was used to compare groups, and LSD- *t*-test was used for statistical analysis of multiple comparisons between groups.

## Results

### The three main SCFAs inhibited dsDNA production

**Fig. 1:**
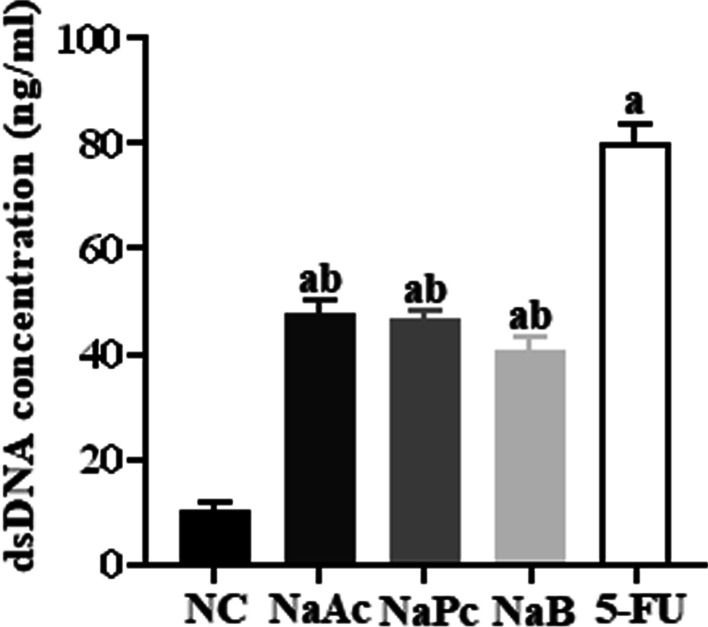
The dsDNA concentration in cell supernatant (n = 3). 1: NC group; 2: NaAc group; 3: NaPc group; 4: NaB group; 5:5-FU group; a: compared with NC group, *P* < 0.05; b: compared with 5-FU group, *P* < 0.05.

When THP-1 cells were treated with 5-FU for 24 h, compared with the NC group, the concentration of dsDNA in the supernatant in the 5-FU group was significantly increased (*P* < 0.05). Compared with the 5-FU group, the concentrations of dsDNA in NaAc, NaPc, NaB groups were decreased significantly (Fig. 1).

### The three main SCFAs inhibited ROS expression in THP-1 cells and Caco-2 cells

**Fig. 2:**
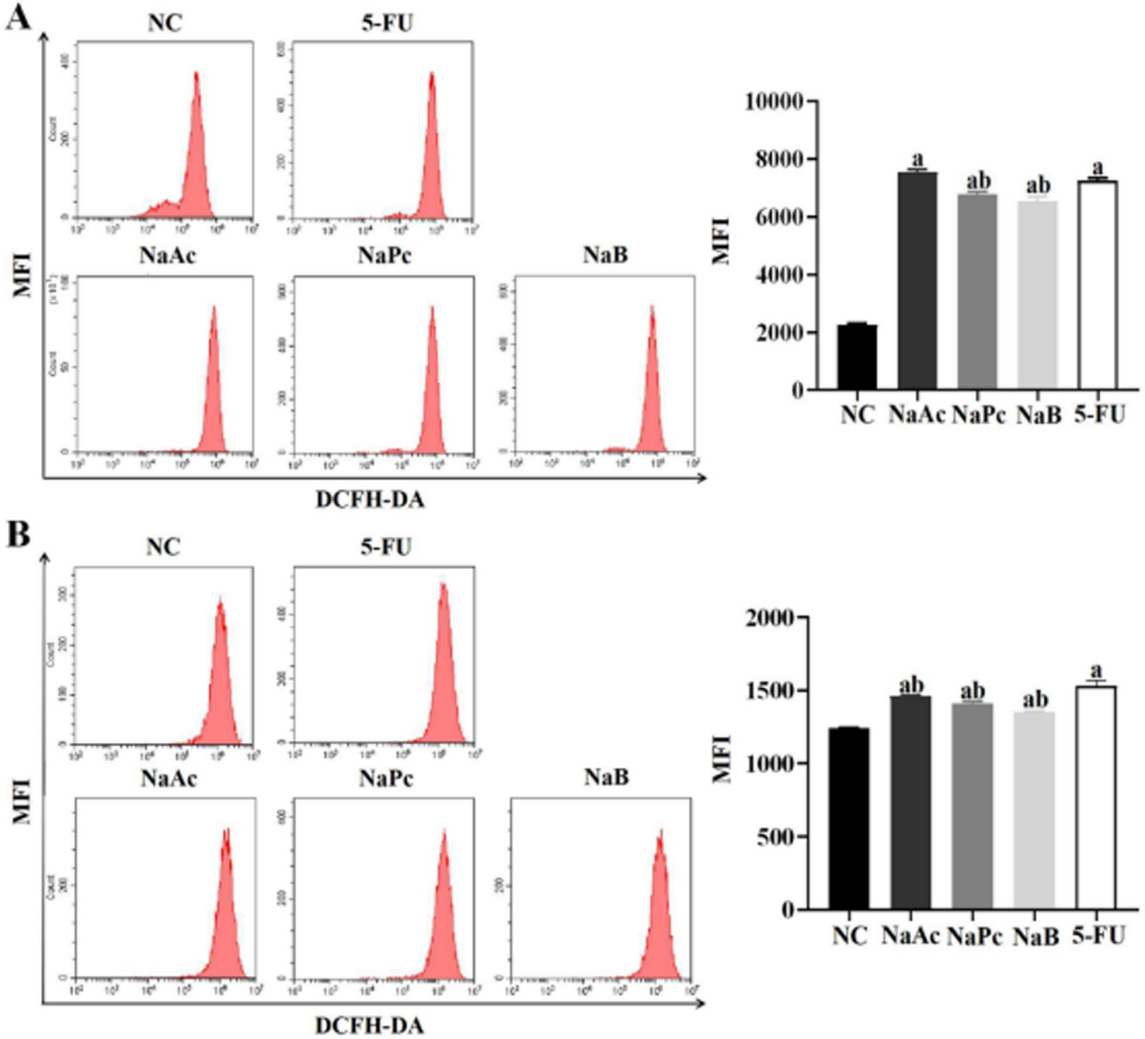
The ROS expression in THP-1 cells and Caco-2 cells (n = 3). **A**: THP-1 cell; **B**: Caco-2 cell; 1: NC group; 2: NaAc group; 3: NaPc group; 4: NaB group; 5:5-FU group; a: *P* < 0.05, compared with control group; b: *P* < 0.05, compared with 5-FU group

The flow cytometry method was used to determine ROS expression in THP-1 cells and Caco-2 cells (Fig. 2). Compared with the NC group, ROS expression in the 5-FU group in THP-1 cells and Caco-2 cells increased significantly (*P* < 0.05). Compared with the 5-FU group, the expressions of ROS in NaPc and NaB groups in THP-1 cells were decreased significantly (*P* < 0.05), and in NaAc, NaPc, NaB groups in Caco-2 cells were significantly reduced (*P* < 0.05).

### The three main SCFAs inhibited inflammatory factors expressions in THP-1 cells and Caco-2 cells

**Fig. 3:**
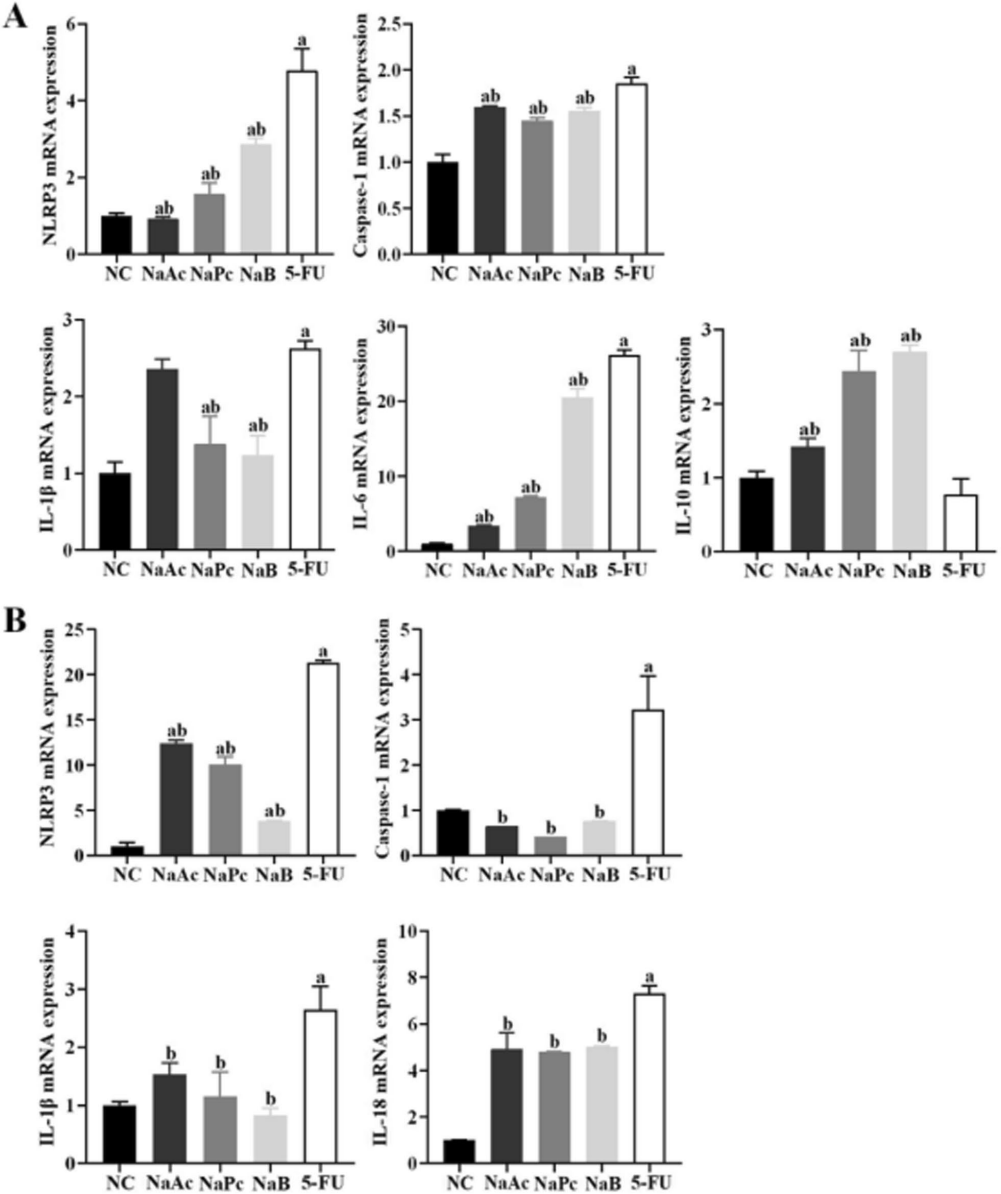
The mRNA expressions of NLRP3, Caspase-1, IL-1β, IL-6 and IL-10 in THP-1 cell and Caco-2 cell (n = 3). **A**: THP-1 cell; **B**: Caco-2 cell; 1: NC group; 2: NaAc group; 3: NaPc group; 4: NaB group; 5: 5-FU group; a: compared with NC group, *P* < 0.05; b: compared with 5-FU group, *P* < 0.05

In THP-1 cells, the expressions of NLRP3, Caspase-1, IL-1β and IL-6 mRNA in the 5-FU group were significantly higher than those in the NC group (*P* < 0.05). Compared with the 5-FU group, the mRNA expressions of IL-1β in NaPc and NaB groups decreased significantly (*P* < 0.05), the mRNA expressions of NLRP3, Caspase-1 and IL-6 in NaAc, NaPc, NaB groups decreased significantly (*P* < 0.05), and the mRNA expression of IL-10 in NaAc, NaPc, NaB groups increased significantly (*P* < 0.05) (Fig. 3A).

In Caco-2 cells, compared with the NC group, the mRNA expression of NLRP3, Caspase-1, IL-1β and IL-18 in the 5-FU group were significantly increased (*P* < 0.05). Compared with the 5-FU group, the mRNA expression of NLRP3, Caspase-1, IL-1β, and IL-18 can be inhibited when treated with NaAc, NaPc and NaB, respectively (*P* < 0.05) (Fig. 3B).

### NaB inhibited NF-κB p65 nuclear translocation in THP-1 cells

**Fig. 4:**
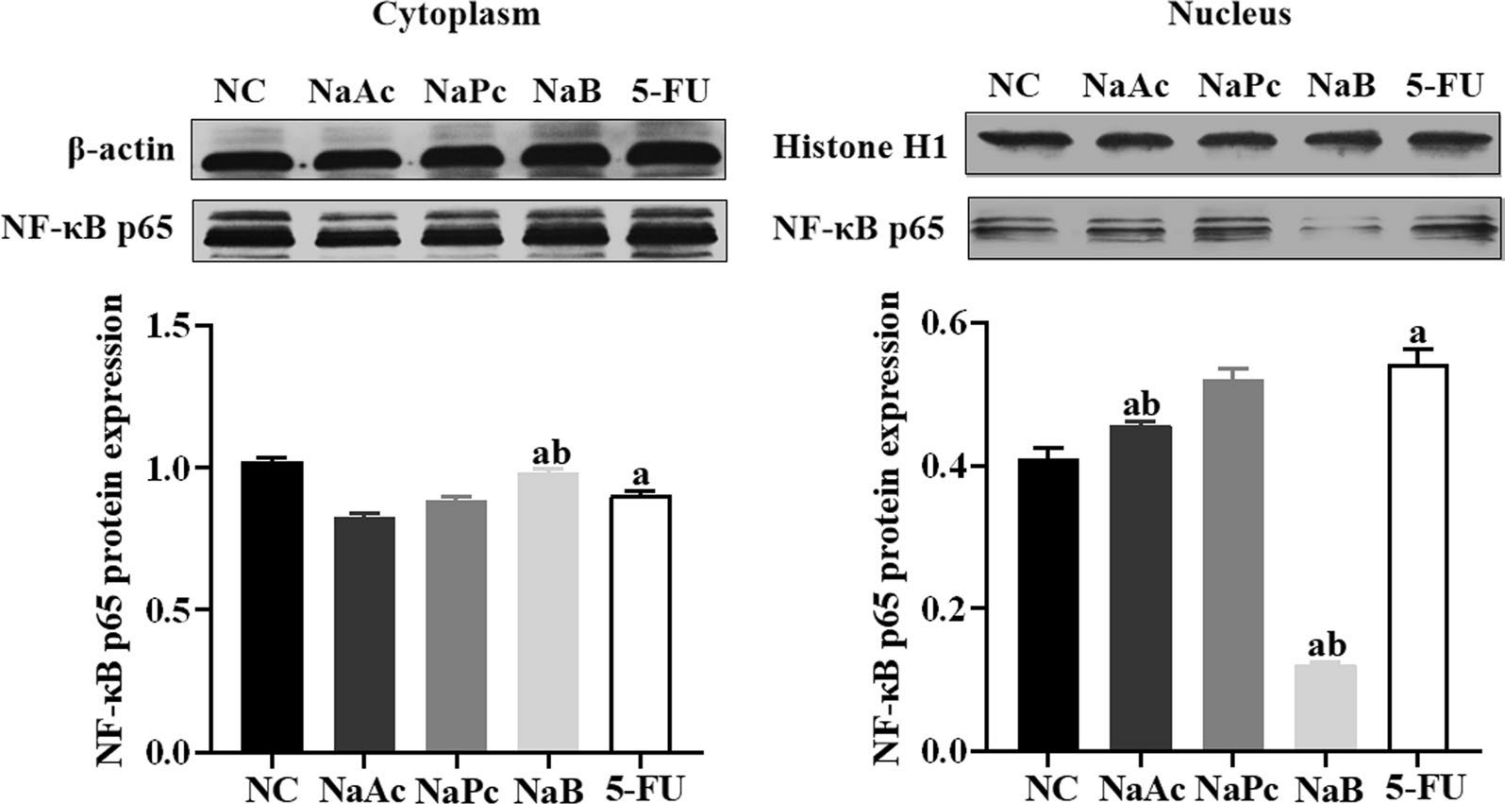
The expression of NF-κB p65 cytoplasm and nucleus in THP-1 cell (n = 3). 1: NC group; 2: NaAc group; 3: NaPc group; 4: NaB group; 5: 5-FU group; a: compared with NC group, *P* < 0.05; b: compared with 5-FU group, *P* < 0.05.

When NF-κB p65 protein in cytoplasm entered the nucleus, the NF-κB pathway was activated. The expressions of NF-κB p65 in the nucleus and cytoplasm of THP-1 cells were determined by Western blotting (Fig. 4). Compared with the NC group, the expression of NF-κB p65 in the nucleus was significantly increased, and the expression in plasma was significantly decreased in the 5-FU group. Compared with the 5-FU group, the NF-κB p65 expressions in the cytoplasm was boosted considerably in NaB group (*P* < 0.05), and the expression in the nucleus was decreased significantly in the NaAc group and NaB group (*P* < 0.05).

### The three main SCFAs inhibited the expression of LC3-II and Beclin-1 in THP-1 cells

**Fig. 5:**
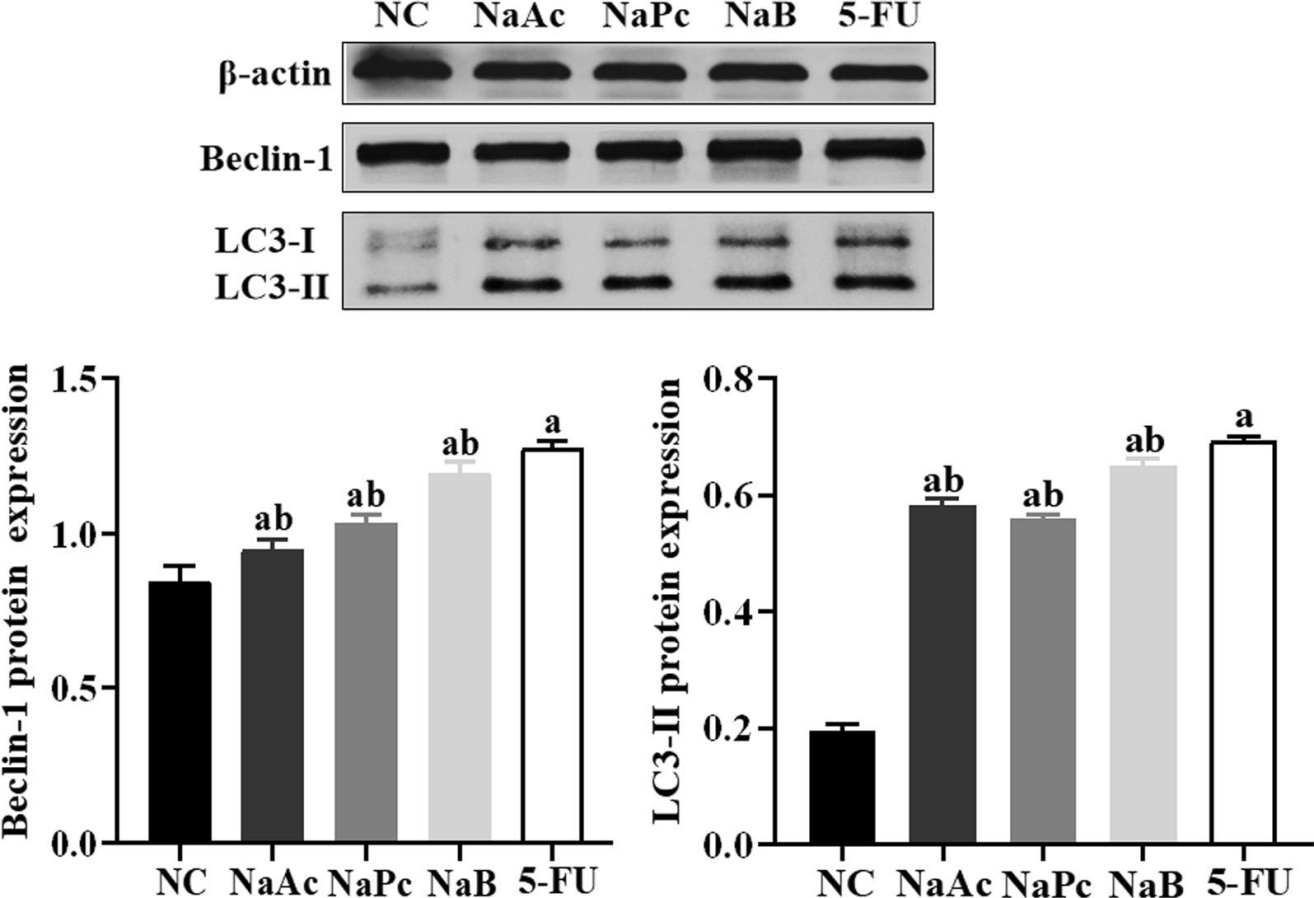
The expression of LC3-II and Beclin-1 in THP-1 cells (n = 3). 1: NC group, 2: NaAc group; 3: NaPc group; 4: NaB group; 5: 5-FU group; a: *P* < 0.05, compared with NC group; b: *P* < 0.05, compared with 5-FU group

When the expression of autophagy-related proteins LC-3-II and Beclin-1 increased, the degree of autophagy also increased. The expression of LC3-II and Beclin-1 in THP-1 cells was detected by Western blotting assay (Fig. 5). Compared with the NC group, the expressions of LC3-II and Beclin-1 were significantly increased in the 5-FU group (*P* < 0.05). Compared with the 5-FU group, the expressions of LC3-II and Beclin-1 were significantly decreased in NaAc, NaPc, NaB groups (*P* < 0.05).

### The three main SCFAs inhibited the expressions of Occludin MUC2 in Caco-2 cells

**Fig. 6:**
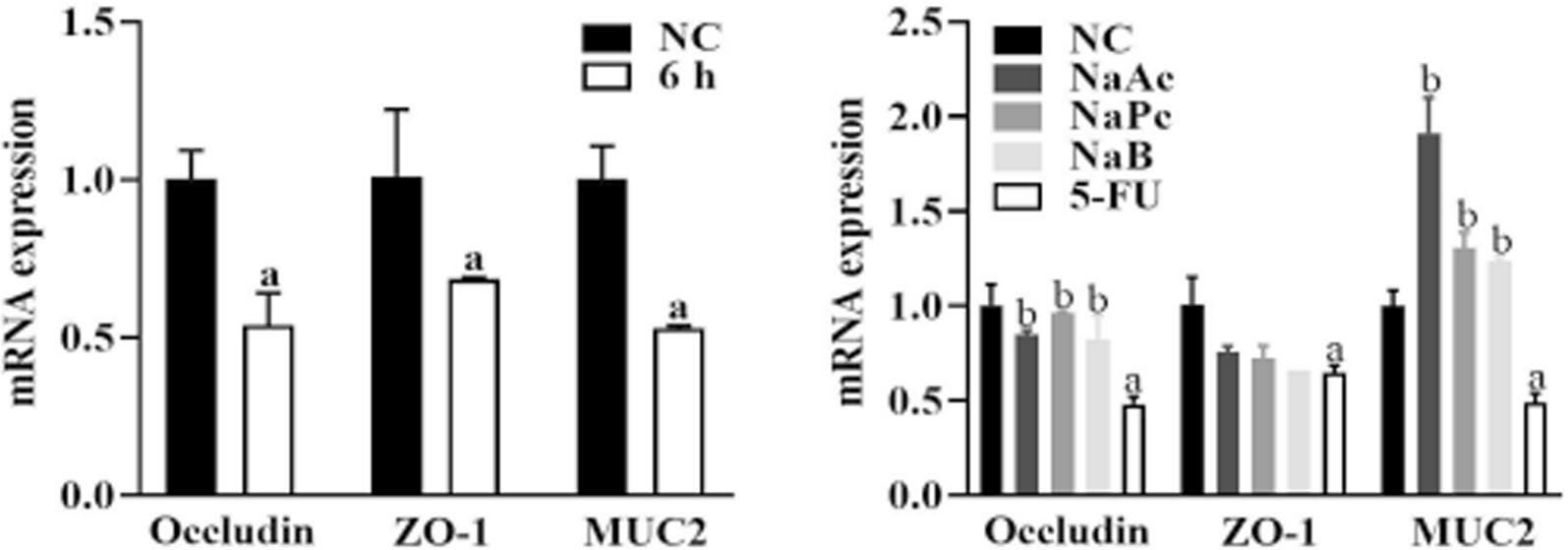
The mRNA expressions of ZO-1, Occludin and MUC2 in Caco-2 cells (n = 3). 1: NC group, 2: NaAc group; 3: NaPc group; 4: NaB group; 5: 5-FU group; a: *P* < 0.05, compared with NC group; b: *P* < 0.05, compared with 5-FU group

Tight junction proteins ZO-1 and Occludin together with MUC2 from the mucosal barrier. Compared with the NC group, the mRNA expression of Occludin, ZO-1, MUC2 in the 5-FU group decreased significantly at 6 h (*P* < 0.05). When pretreated with NaAc, NaPc and NaB, compared with the 5-FU group, the mRNA expressions of Occludin and MUC2 were significantly (*P* < 0.05), and ZO-1 mRNA expression had no significant difference (*P* > 0.05) (Fig. 6).

### *L. rhamnosus* can attenuate weight loss

**Fig. 7:**
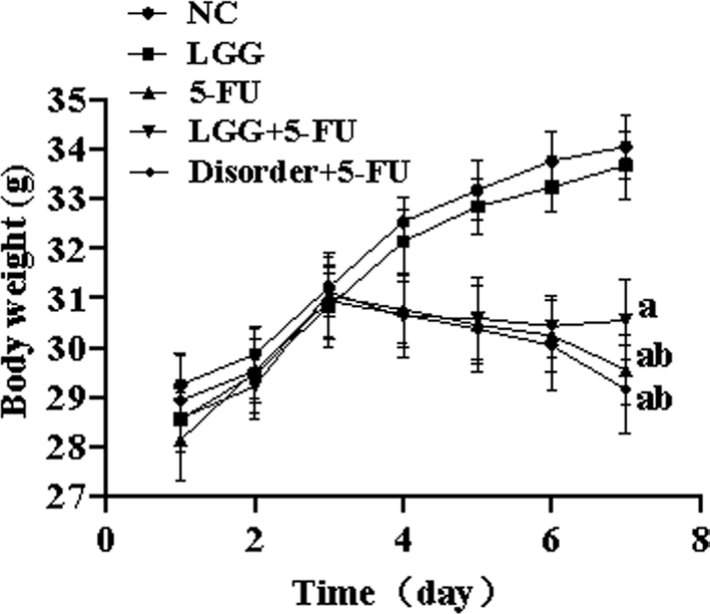
Bodyweight changes in mice. a: *P* < 0.05, compared with NC group; b: *P* < 0.05, compared with LGG + 5-FU group

Bodyweight changes of mice were recorded for 7 days, NC and LGG groups maintained steady weight growth during the experimental period. Mice in 5-FU, disorder + 5-FU and LGG + 5-FU group with an intraperitoneal injection of 5-FU showed a significant loss in body weight on the following day and a constant weight loss during drug administration (*P* < 0.05). Mice in the seventh-day LGG + 5-FU group weighed significantly more than those in 5-FU and disorder + 5-FU group (*P* < 0.05) (Fig. 7).

### *L. rhamnosus* increased the concentration of NaAc, NaPc and NaB in faeces

**Fig. 8:**
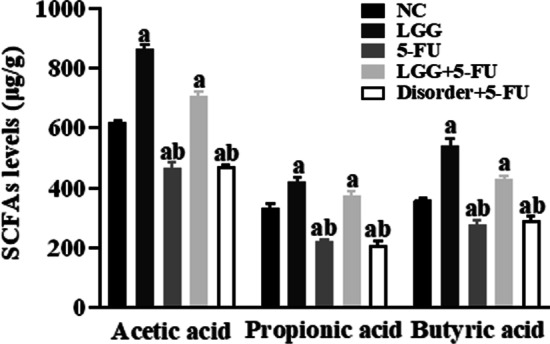
Analysis of SCFAs content in mouse faeces by gas chromatography. a: *P* < 0.05, compared with NC group; b: *P* < 0.05, compared with LGG + 5-FU group

Compared with the NC group, the concentrations of NaAc, NaPc and NaB in faeces in the 5-FU and disorder + 5-FU group were significantly decreased (*P* < 0.05). After oral administration of *L. rhamnosus*, the concentrations of NaAc, NaPc and NaB in faeces were considerably higher than those of the NC, 5-FU and disorder + 5-FU group (*P* < 0.05). The results showed that *L. rhamnoides* could significantly increase the production of three major SCFAs in the colon (Fig. 8).

### *L. rhamnosus* increased the expressions of ZO-1 and Occludin in the small intestinal mucosa

**Fig. 9:**
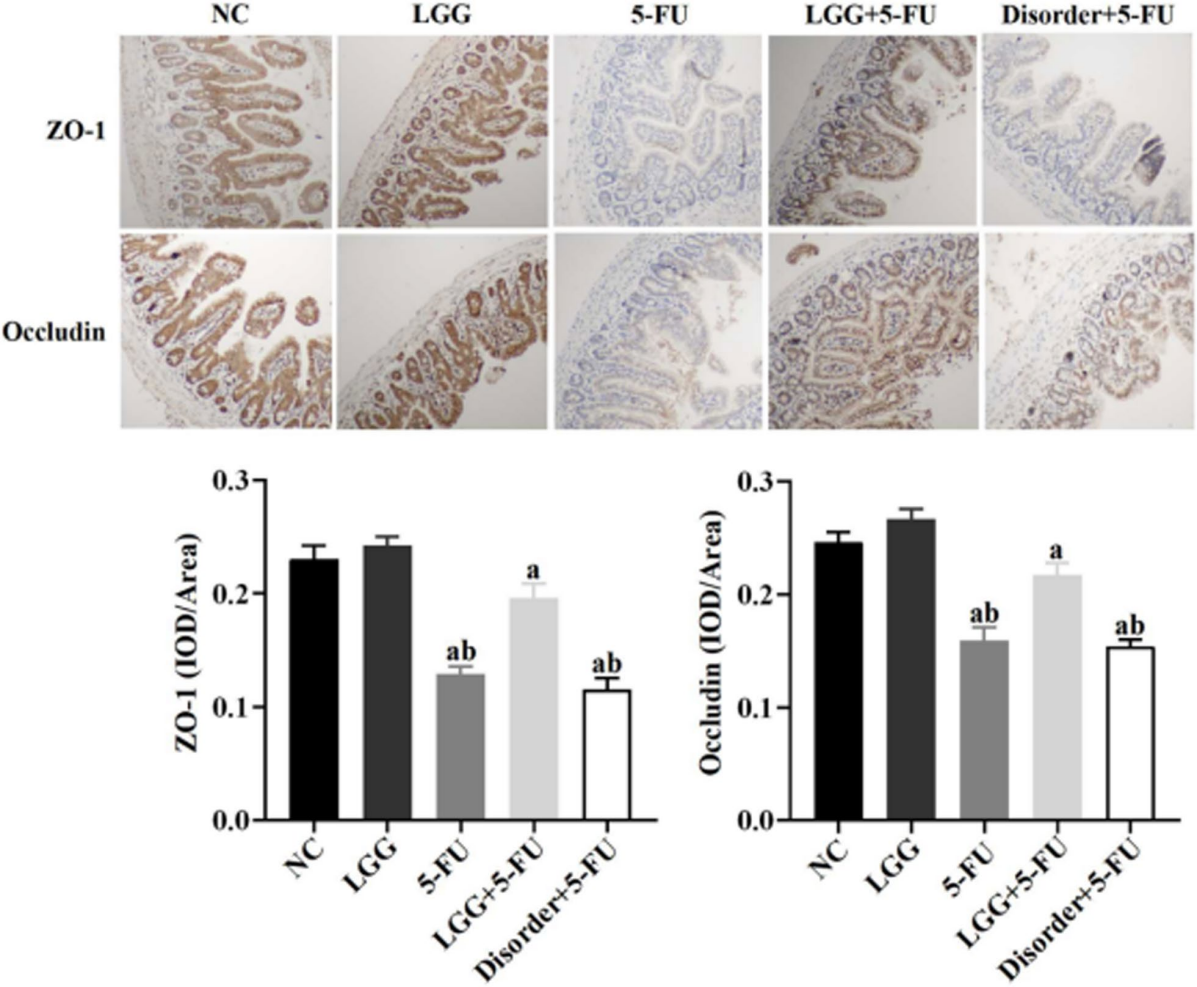
The expressions of ZO-1 and Occludin detected by IHC. a: *P* < 0.05, compared with NC group; b: *P* < 0.05, compared with LGG + 5-FU group

In mice, small intestine tissue immunohistochemical (IHC) analysis, compared with NC group, the expressions of ZO-1 and Occludin in 5-FU and disorder + 5-FU group were significantly decreased (*P* < 0.05). Compared with the 5-FU and disorder + 5-FU group, the expressions of ZO-1 and Occludin in the LGG + 5-FU group were significantly increased (*P* < 0.05) (Fig. 9).

### *L. rhamnosus* increased the expression of proinflammatory cytokines in serum

**Fig. 10:**
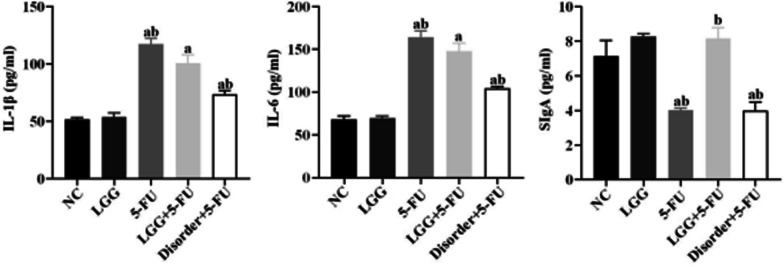
The detection of IL-1 β, IL-6 and sIgA in serum of mice by ELISA. a: *P* < 0.05, compared with NC group; b: *P* < 0.05, compared with LGG + 5-FU group

The concentrations of serum IL-1β and IL-6 in the 5-FU group, LGG + 5-FU group and disorder + 5-FU group, were higher than that in the NC group (*P* < 0.05), and the concentrations of serum IL-1β and IL-6 in the LGG + 5-FU group were lower than 5-FU group (*P* < 0.05). The concentration of serum IgA in the 5-FU group, disorder + 5-FU group, was lower than the NC group and LGG + 5-FU group (*P* < 0.05) (Fig. 10).

### *L. rhamnosus* modulated the expression of NLRP3 and proinflammatory cytokines in splenocytes

**Fig. 11:**
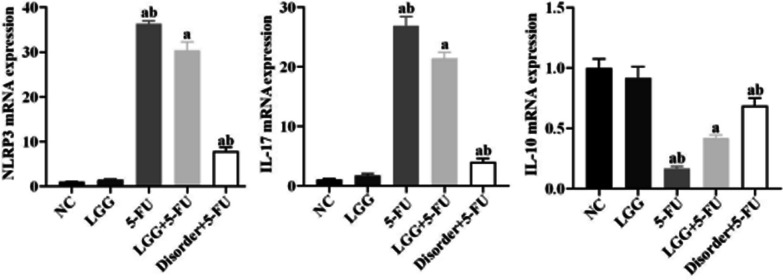
The expressions of NLRP3, Caspase-1, IL-17 and IL-10 mRNA were detected by qRT-PCR. a: *P* < 0.05, compared with NC group; b: *P* < 0.05, compared with LGG + 5-FU group

Compared with the NC group, the expressions of NLRP3 and IL-17 mRNA in the spleen in the 5-FU group, LGG + 5-FU group and disorder + 5-FU group were significantly increased (*P* < 0.05), and the expression of IL-10 mRNA was significantly decreased (*P* < 0.05). Compared with the 5-FU group, the expressions of NLRP3 and IL-17 mRNA were reduced considerably (*P* < 0.05) and the expression of IL-10 mRNA was increased considerably in the LGG + 5-FU group (*P* < 0.05) (Fig. 11).

## Discussion

It is known that 5-FU not only interferes with DNA synthesis of tumor cells but also interferes with DNA synthesis of normal tissue cells, resulting in the production of a large number of reactive oxygen species and cell death and the release of a large number of double-stranded DNA (dsDNA) [12]. We found that 5-FU leads to the cell damage and releases a large amount of dsDNA in the cell supernatant. As an important damage-associated molecular pattern (DAMPs), the dsDNA is critical in inducing inflammatory response and autoimmune injury [13]. NLRP3 is the essential member of the inflammasome family, which can be activated by bacterial toxins, ATP, ROS, urea crystallization and other pathogens and dangerous signal molecules in the body. NLRP3 is an important factor in antigenic immunity and inducing inflammatory diseases [14]. In the absence of intestinal infection, the expression of NLRP3 in intestinal mucosal epithelial cells and macrophages is deficient. Once activated, the expression is up-regulated in intestinal immune cells. Then the assembly of activated protease caspase-1 promotes the cleavage and maturation of IL-1β and IL-18 precursors, triggering a severe inflammatory response [15]. Our study found that 5-FU can up-regulate the expression of reactive oxygen species and dsDNA in THP-1 cells, promote the entry of NF-κB p65 protein into the cell nucleus, activate NLRP3 inflammasome, and finally increase the expression level of various proinflammatory factors. It is suggested that 5-FU can activate the NLRP3 inflammasome in THP-1 cells through ROS/NF-κB/NLRP3 signal pathway and then trigger a severe inflammatory response.

Autophagy is a process of cell self-degradation. It is usually activated by hunger or other stresses such as hypoxia, reactive oxygen species, DNA damage and so on, which is of great significance for maintaining homeostasis [16, 17]. *Beclin-1* gene is the first confirmed mammalian autophagy regulatory gene, and its coding product Beclin-1 is a marker protein of autophagy [18]. LC3-II is the only autophagy-associated protein located on the autophagy membrane, and its content is proportional to the number of autophagy vesicles [19]. Macrophages are an essential part of host innate immunity. Autophagy occurs in macrophages, and the secretions of its proinflammatory cytokines are also reduced, and autophagy can inhibit the activation of Caspase-1 in the NLRP3 inflammasome. However, the excessive autophagy in macrophages leads to decreased host innate immunity and increases the probability of opportunistic infection. To ensure that the inflammatory response of macrophages is reduced while still maintaining a certain level of immunity is the current dilemma of tumor treatment [20, 21]. This study found that all three kinds of SCFAs could inhibit the inflammatory response of THP-1 cells in varying degrees, reduce the level of cellular inflammation, and reduce the level of autophagy of THP-1 maintained the number of cells.

SCFAs, the main product of anaerobic bacteria fermentation, is closely related to the regulation of the intestinal immune system. We found that *L. rhamnoides* could significantly increase the concentration of SCFAs in the intestine of mice. In the human intestinal tract, the concentration of SCFAs can reach 70–140 mmol/L, and is rapidly absorbed by the blood, mainly sodium acetate, sodium propionate and sodium butyrate [22]. SCFAs produced by fermentation in the intestinal tract can provide energy for the proliferation of intestinal mucosal epithelial cells, promote cell metabolism and growth, and regulate the composition of human intestinal flora and reduce the growth of harmful bacteria and prevent intestinal dysfunction [23]. More importantly, SCFAs can inhibit the release of various proinflammatory factors by immune cells, inhibit the excessive intestinal inflammatory response, and reduce intestinal mucosal damage in patients with colitis [24, 25]. Previous studies have shown that three main SCFAs (sodium acetate, sodium propionate and sodium butyrate) can reduce proinflammatory factors from immune cells, inhibit the inflammatory response, and promote the production of anti-inflammatory cytokines IL-10 at lower concentrations (1–1200 μmol/L). At the same time, acetate and butyrate can also exert anti-inflammatory effects by inhibiting the activation of the NF-κB signal pathway [26]. The results of this study in vitro are consistent with the above results. Sodium propionate and sodium butyrate can significantly down-regulate the expression of proinflammatory factor IL-1β in THP-1 cells. The three kinds of SCFAs can significantly reduce the expression of proinflammatory factor IL-6 and up-regulate the expression of anti-inflammatory factor IL-10. Sodium propionate can also reduce the production of reactive oxygen species, while sodium acetate and sodium butyrate can significantly inhibit the translocation of NF-κB p65 protein into the nucleus. Thus inhibit the activation of NLRP3 inflammasome and reduce the inflammatory response. In addition, this study also found that the three kinds of SCFAs could also inhibit the activation of NLRP3 inflammasome in Caco-2 cells and inhibit the expression of inflammatory cytokines IL-18. However, in the animal model study, the increasing SCFAs in the intestinal tract of mice reduced the expression of inflammatory cytokines in mice serum and spleen cells.

The intestinal mucosal barrier is composed of mechanical barrier, biological barrier, immune barrier and chemical barrier, in which the mechanical barrier is mainly composed of the mucous layer on the surface of the intestinal mucosa, the tight junction between closely arranged epithelial cells and epithelial cells, and mucin MUC2 plays a vital role in the formation of a mucous layer on the surface of intestinal epithelium [27]. We found that 5-FU stimulation of Caco-2 cells and mouse intestinal epithelial cells could reduce the expression of mucin MUC2, thus destroying the integrity of the intestinal mucosal barrier. The three kinds of SCFAs pre-treatment could significantly increase the expression of mucin MUC2 to repair the intestinal mucosal barrier. The tight junction regulates intestinal paracellular permeability. Intestinal epithelial cells maintain the integrity and tight junction of intestinal epithelium by expressing proteins such as atresia zone protein ZO-1 and tight junction protein occludin to resist the invasion of flora and external pathogens [28]. Previous studies have found that 5-FU significantly increases intestinal permeability by reducing the expression of Occludin and claudin-1 and increases the penetration of bacteria and proinflammatory cytokines, resulting in inflammatory reaction or aggravation [29]. The results of this study are consistent with the above results; we found that the expression of ZO-1 and Occludin in Caco-2 cells and mouse intestinal epithelial cells decreased significantly, and intestinal permeability increased significantly. All the three kinds of SCFAs significantly up-regulated the expression of Occludin, thus reducing intestinal permeability and protecting the integrity of intestinal mucosal tight junction.

SIgA, which is secreted and assembled by plasma cells and intestinal epithelial cells in intestinal lamina propria, is an integral part of intestinal mucosal immunity and the first line of defense against bacterial adhesion and colonization in the intestinal mucosa. After chemotherapy with 5-FU, the function of secretory immunoglobulin produced by the intestinal tract was significantly inhibited, characterized by a decrease in sIgA content [30]. The results showed that 5-FU could destroy mice's intestinal mucosal immune barrier, which showed that the content of sIgA in the serum of mice decreased significantly. In contrast, the content of sIgA in the serum of mice increased significantly after oral administration of *L. rhamnoides*, suggesting that the intestinal mucosal immune barrier was effectively protected.

In summary, in the in vivo experiment, we significantly increased the contents of acetic acid, propionic acid and butyric acid by oral administration of *L. rhamnoides*, and *L. rhamnlidate* might inhibit the inflammation in mice by increasing the production of SCFAs. It can significantly protect tight junction integrity between intestinal mucosal epithelial cells. In vitro, we found that the three kinds of SCFAs could inhibit the expression of reactive oxygen species and the activation of NLRP3 inflammatory bodies to inhibit the expression of inflammatory factors, protect the integrity of the mucosal barrier and reduce autophagy. Acetate and butyrate could significantly inhibit the translocation of NF-κB p65 protein into the nucleus, thus decreasing the inflammatory reaction. All three kinds of SCFAs can inhibit cellular inflammation in varying degrees, but the specific mechanisms are not entirely the same, and the particular reasons need to be further discussed. The molecular mechanism of the 5-FU-induced mucosal barrier integrity damage induced by SCFAs is unclear. The effect of activation of NLRP3 inflammasome on mucosal barrier integrity can be further studied.

## Data Availability

All data generated or analyzed during the current study available from the corresponding author on reasonable request.

## Abbreviations

SCFAs: Short-chain fatty acids
5-FU: Fluorouracil
NLRP3: NLR family pyrin domain containing 3
MUC2: Mucin-2
NF-κB: Nuclear factor kappa-B
DAMPs: Damage-associated molecular patterns
LC3-II: Microtubule-associated proteins light chain 3 II
ROS: Ractive oxygen species
NaAc: Sodium acetate
NaPc: Sodium propionate
NaB: Sodium butyrate
THP-1 cell: Human mononuclear macrophage cells
Caco-2 cell: Colorectal adenocarcinoma cells
